# Supplementary material for: Pseudomonads coordinate innate defense against viruses and bacteria with a single regulatory system
Source: bioRxiv. 2025 Feb 27:2025.02.26.640152. Preprint. [Version 1] doi: 10.1101/2025.02.26.640152 (PMC11888443; doi:10.1101/2025.02.26.640152)
Supplement: Supplement 3 [file media-3.pdf]

Table S3: Summary of phage defense systems, related to Figure 2

Organism	Locus Tag	Defense System	Gene	GRP fold activation	Adj. p-value
P. aeruginosa	PA0574	SoFic	SoFic	NA	NA
	PA0715	Retron_I-B	RT_I-B	1.005	0.977
	PA0716	Retron_I-B	ATPase-Toprim_I-B	0.352	0.414
	PA1370	PDC-S39	PDC-S39	1.433	0.423
	PA1371	Helicase-DUF2290	DUF2290	NA	NA
	PA1372	Helicase-DUF2290	Helicase	0.896	0.185
	PA1671	PD-T4-6	PD-T4-6	NA	NA
	PA1935	Gabija	GajB	NA	NA
	PA1939	Gabija	GajA	4.940	8.09E-03
	PA2732	RM_type_I	REase_I	1.11	0.325
	PA2734	RM_type_I	Specificity_I	0.659	0.0432
	PA2735	RM_type_I	MTase_I	0.735	0.0443
P. protegens	PFL_1011	PDC-S04	PDC-S04	0.802	NA
	PFL_1398	PDC-S21	PDC-S21	0.914	0.320
	PFL_2023	PARIS_I	AriA	172.9	4.75E-03
	PFL_2561	PDC-S02	PDC-S02	NA	NA
	PFL_2676	PDC-S12	PDC-S12	0.989	0.923
	PFL_2962	Septu_type_I	PtuB1	NA	NA
	PFL_2963	Septu_type_I	PtuA1	1.035	0.950
	PFL_2964	DMS_other	Specificity_I	1.796	0.150
	PFL_2965	DMS_other	MTase_I	3.028	0.0212
	PFL_3013	Druantia_type_II	DruM2	5.100	0.0734
	PFL_3014	Druantia_type_II	DruF2	1.608	NA
	PFL_3015	Druantia_type_II	DruG2	5.454	0.0095
	PFL_3016	Druantia_type_II	DruE2	16.903	3.87E-06
	PFL_4270	PDC-S08	PDC-S08	NA	NA
	PFL_6252	BstA	BstA	29.729	4.86E-05
P. fluorescens	PFLU3_01560	PDC-S21	PDC-S21	0.449	0.111
	PFLU3_04460	RM_type_IV	mREase_IV	7.926	1.06E-05
	PFLU3_11630	SoFic	SoFic	0.687	0.315
	PFLU3_12200	Hachiman_type_I	HamA1	6.673	1.409E-04
	PFLU3_12210	Hachiman_type_I	HamB1	NA	NA
	PFLU3_12220	DRT_class_III	RT_UG5-nitrilase	NA	NA
	PFLU3_12490	PDC-S04	PDC-S04	NA	NA
	PFLU3_14030	SoFic	SoFic	NA	NA
	PFLU3_18670	AbiE	AbiEii	NA	NA
	PFLU3_18680	AbiE	AbiEi	NA	NA
	PFLU3_21860	Kiwa	KwaB	20.798	1.478E-04
	PFLU3_21870	Kiwa	KwaA	NA	NA
	PFLU3_21970	Druantia_type_III	DruE3	2.034	3.956E-04
	PFLU3_21980	Druantia_type_III	DruH3	NA	NA
	PFLU3_21990	RM_type_I	REase_I	1.354	0.405

Organism	Locus Tag	Defense System	Gene	GRP fold activation	Adj. p-value
P. fluorescens	PFLU3_22000	PDC-S14	PDC-S14	0.402	0.0209
	PFLU3_22010	RM_type_I	Specificity_I	0.456	0.0307
	PFLU3_22030	RM_type_I	MTase_I	0.701	2.779E-03
	PFLU3_28130	PDC-S24	PDC-S24	0.606	0.250
	PFLU3_30380	Bunzi	BnzA	5.815	9.754E-04
	PFLU3_30390	Bunzi	BnzB	0.967	0.967
	PFLU3_31980	Shango	SngC	NA	NA
	PFLU3_31990	Shango	3S	23.817	5.151E-04
	PFLU3_32000	Shango	SngA	NA	NA
	PFLU3_37090	DRT_class_III	Drt1a	NA	NA
	PFLU3_37260	DMS_other	BrxHI	NA	NA
	PFLU3_37300	DMS_other	DrmC	NA	NA
	PFLU3_41100	PD-T7-4	PD-T7-4	7.004	2.48E-05
	PFLU3_42830	PD-T4-6	PD-T4-6	NA	NA
	PFLU3_45960	PDC-S08	PDC-S08	NA	NA
	PFLU3_49280	SoFic	SoFic	NA	NA
	PFLU3_55230	PDC-S12	PDC-S12	0.762	0.185
	PFLU3_56560	Mokosh_TypeII	MkoC	9.009	1.817E-04
P. putida	PP_0049	PD-T7-1	PD-T7-1	NA	NA
	PP_1161	PDC-S12	PDC-S12	0.851	0.204
	PP_1406	PDC-S21	PDC-S21	0.871	0.528
	PP_2277	PDC-S58	PDC-S58	NA	NA
	PP_2531	PDC-S08	PDC-S08	NA	NA
	PP_3680	Gabija	GajA	6.994	7.915E-04
	PP_3681	Gabija	GajB	NA	NA
	PP_3692	PDC-S06	PDC-S06	NA	NA
	PP_3694	Wadjet_type_I	JetD1	NA	NA
	PP_3695	Wadjet_type_I	JetD1	NA	NA
	PP_3696	Wadjet_type_I	JetC1	NA	NA
	PP_3697	Wadjet_type_I	JetC1	NA	NA
	PP_3698	Wadjet_type_I	JetA1	NA	NA
	PP_3708	PDC-S64	PDC-S64	NA	NA
	PP_3988	RM_Type_II	Type_II_Rease	3.883	5.233E-03
	PP_3989	RM_Type_II	Type_II_Mtase	1.023	0.768
	PP_4447	GAO_20	DUF4297	2.409	0.0397
	PP_4448	GAO_20	HerA	8.982	2.41E-05
	PP_4740	RM_type_I	REase_I	4.544	0.0121
	PP_4741	RM_type_I	MTase_I	0.994	0.949
	PP_4742	RM_type_I	Specificity_I	0.995	0.973
	PP_5622	Wadjet_type_I	JetB1	NA	NA
	PP_5643	PDC-M53	PDC-M53A	2.768	1.721E-04
	PP_5644	PDC-M53	PDC-M53B	NA	NA

Table S4: Strains and plasmids used in this study

**Strains**		
Organism	Genotype	Source
P. aeruginosa PAO1	parental	1
	Δ*gacS* (PA0928)	2
	Δ*retS* (PA4856)	3
P. protegens Pf-5	parental	4
	Δ*gacS* (PFL_4451)	This study
	Δ*retS* (PFL_0664)	This study
	ΔPFL_5124	This study
	ΔPARIS (PFL_2023 and downstream ORF)	This study
	Δ*bstA* (PFL_6252)	This study
	ΔPARIS attTn7::AraE-AraC-pBad-PARIS	This study
	Δ*bstA* attTn7::AraE-AraC-pBad-*bstA*	This study
	Δ*phlD* (PFL_5957)	This study
	ΔOBC4 (PFL_5483 - PFL_5495)	This study
P. fluorescens 2-79	parental	5
	Δ*gacS* (PFLU3_03780)	This study
	Δ*retS* (PFLU3_30700)	This study
P. putida KT2440	parental	6
	Δ*gacS* (PP_1650)	This study
	Δ*retS* (PP_4824)	This study
P. putida IsoF	parental	7
	Δ*gacS* (PisoF_00466)	This study
V. parahaemolyticus RIMD 2210633	parental	8
	Δ*gacA* (VP1945)	This study
E. cloacae ATCC 13047	parental	9
	Δ*tssM* (ECL_RS07530)	10
B. thailandensis E264	parental	11
	Δ*tssM-1* (BTH_I2954)	2
L. enzymogenes C3-1	parental	12
	Δ*virD4* (GLE_2798)	This study
E. coli MG1655	parental	13
**Plasmids**		
Plasmid	Utility	Source
pDMB003_pEXG2_C3_virD4_ko	*L. enzymogenes virD4* deletion allele	This study
pDMB005_pEXG2_Pf-5_gacS_ko	*P. protegens gacS* deletion allele	This study
pDMB006_pEXG2_KT2440_gacS_ko	*P. putida* KT2440 Δ*gacS* deletion allele	This study
pDMB009_pEXG2_Pf-5_retS_ko	*P. protegens* Δ*retS* deletion allele	This study
pDMB010_pEXG2_KT2440_retS_ko	*P. putida* KT2440 Δ*retS* deletion allele	This study

pDMB015_pRE112_Vp_gacA_ko	*V. parahaemolyticus* Δ*gacA* deletion allele	This study
pDMB038_pEXG2_Pf5_OBC4_ko	*P. protegens* ΔOBC4 deletion allele	This study
pDMB041_pEXG2_2-79_gacS_ko	*P. fluorescens* Δ*gacS* deletion allele	This study
pDMB043_pEXG2_IsoF_gacS_ko	*P. putida* IsoF Δ*gacS* deletion allele	This study
pDMB055_pEXG2_Pf5_phlD_ko	*P. protegens* Δ*phlD* deletion allele	This study
pDMB080_pEXG2_Pf5_PARIS_ko	*P. protegens* ΔPARIS deletion allele	This study
pDMB082_pEXG2_Pf5_BstA_ko	*P. protegens* Δ*bstA* deletion allele	This study
pDMB090_pUC18T-miniTn7T-araC-pBad-PARIS-araE	*P. protegens* PARIS complementation	This study
pDMB093_pUC18T-miniTn7T-araC-pBad-aba_bstA-araE	*P. protegens bstA* complementation	This study
pEXG2	Allelic exchange vector	14
pRK2013	tri-parental mating helper plasmid	15
pTNS3	tri-parental mating helper plasmid	16
pUC18T-miniTn7T-araC-pBad-PARIS-araE	Arabinose-inducible expression from attTn7 neutral site	17

Supplementary References

1. Stover, C.K., Pham, X.Q., Erwin, A.L., Mizoguchi, S.D., Warrener, P., Hickey, M.J., Brinkman, F.S.L., Hufnagle, W.O., Kowalik, D.J., Lagrou, M., et al. (2000). Complete genome sequence of Pseudomonas aeruginosa PAO1, an opportunistic pathogen. Nature *406*, 959–964. https://doi.org/10.1038/35023079.

2. LeRoux, M., Kirkpatrick, R.L., Montauti, E.I., Tran, B.Q., Peterson, S.B., Harding, B.N., Whitney, J.C., Russell, A.B., Traxler, B., Goo, Y.A., et al. (2015). Kin cell lysis is a danger signal that activates antibacterial pathways of Pseudomonas aeruginosa. eLife *4*, e05701. https://doi.org/10.7554/eLife.05701.

3. Mougous, J.D., Cuff, M.E., Raunser, S., Shen, A., Zhou, M., Gifford, C.A., Goodman, A.L., Joachimiak, G., Ordoñez, C.L., Lory, S., et al. (2006). A Virulence Locus of Pseudomonas aeruginosa Encodes a Protein Secretion Apparatus. Science *312*, 1526–1530. https://doi.org/10.1126/science.1128393.

4. Paulsen, I.T., Press, C.M., Ravel, J., Kobayashi, D.Y., Myers, G.S.A., Mavrodi, D.V., DeBoy, R.T., Seshadri, R., Ren, Q., Madupu, R., et al. (2005). Complete genome sequence of the plant commensal Pseudomonas fluorescens Pf-5. Nat Biotechnol *23*, 873–878. https://doi.org/10.1038/nbt1110.

5. Nesemann, K., Braus-Stromeyer, S.A., Thuermer, A., Daniel, R., Mavrodi, D.V., Thomashow, L.S., Weller, D.M., and Braus, G.H. (2015). Draft Genome Sequence of the Phenazine-Producing Pseudomonas fluorescens Strain 2-79. Genome Announcements *3*, 10.1128/genomea.00130-15. https://doi.org/10.1128/genomea.00130-15.

6. Bagdasarian, M., and Timmis, K.N. (1982). Host: vector systems for gene cloning in Pseudomonas. Curr Top Microbiol Immunol *96*, 47–67. https://doi.org/10.1007/978-3-642-68315-2_4.

7. Steidle, A., Sigl, K., Schuhegger, R., Ihring, A., Schmid, M., Gantner, S., Stoffels, M., Riedel, K., Givskov, M., Hartmann, A., et al. (2001). Visualization of N-Acylhomoserine Lactone-Mediated Cell-Cell Communication between Bacteria Colonizing the Tomato Rhizosphere. Applied and Environmental Microbiology *67*, 5761–5770. https://doi.org/10.1128/AEM.67.12.5761-5770.2001.

8. Makino, K., Oshima, K., Kurokawa, K., Yokoyama, K., Uda, T., Tagomori, K., Iijima, Y., Najima, M., Nakano, M., Yamashita, A., et al. (2003). Genome sequence of Vibrio parahaemolyticus: a pathogenic mechanism distinct from that of V cholerae. The Lancet *361*, 743–749. https://doi.org/10.1016/S0140-6736(03)12659-1.

9. Ren, Y., Ren, Y., Zhou, Z., Guo, X., Li, Y., Feng, L., and Wang, L. (2010). Complete Genome Sequence of Enterobacter cloacae subsp. cloacae Type Strain ATCC 13047. Journal of Bacteriology *192*, 2463–2464. https://doi.org/10.1128/JB.00067-10.

10. Whitney, J.C., Beck, C.M., Goo, Y.A., Russell, A.B., Harding, B.N., De Leon, J.A., Cunningham, D.A., Tran, B.Q., Low, D.A., Goodlett, D.R., et al. (2014). Genetically distinct pathways guide effector export through the type VI secretion system. Molecular Microbiology *92*, 529–542. https://doi.org/10.1111/mmi.12571.

11. Yu, Y., Kim, H.S., Chua, H.H., Lin, C.H., Sim, S.H., Lin, D., Derr, A., Engels, R., DeShazer, D., Birren, B., et al. (2006). Genomic patterns of pathogen evolution revealed by comparison of Burkholderia pseudomallei, the causative agent of melioidosis, to avirulent Burkholderia thailandensis. BMC Microbiology *6*, 46. https://doi.org/10.1186/1471-2180-6-46.

12. de Bruijn, I., Cheng, X., de Jager, V., Expósito, R.G., Watrous, J., Patel, N., Postma, J., Dorrestein, P.C., Kobayashi, D., and Raaijmakers, J.M. (2015). Comparative genomics and metabolic profiling of the genus Lysobacter. BMC Genomics *16*, 991. https://doi.org/10.1186/s12864-015-2191-z.

13. Blattner, F.R., Plunkett, G., Bloch, C.A., Perna, N.T., Burland, V., Riley, M., Collado-Vides, J., Glasner, J.D., Rode, C.K., Mayhew, G.F., et al. (1997). The Complete Genome Sequence of Escherichia coli K-12. Science *277*, 1453–1462. https://doi.org/10.1126/science.277.5331.1453.

14. Rietsch, A., Vallet-Gely, I., Dove, S.L., and Mekalanos, J.J. (2005). ExsE, a secreted regulator of type III secretion genes in Pseudomonas aeruginosa. Proceedings of the National Academy of Sciences *102*, 8006–8011. https://doi.org/10.1073/pnas.0503005102.

15. Suh, S.-J., Silo-Suh, L.A., and Ohman, D.E. (2004). Development of tools for the genetic manipulation of *Pseudomonas aeruginosa*. Journal of Microbiological Methods *58*, 203–212. https://doi.org/10.1016/j.mimet.2004.03.018.

16. Choi, K.-H., Mima, T., Casart, Y., Rholl, D., Kumar, A., Beacham, I.R., and Schweizer, H.P. (2008). Genetic Tools for Select-Agent-Compliant Manipulation of Burkholderia pseudomallei. Applied and Environmental Microbiology *74*, 1064–1075. https://doi.org/10.1128/AEM.02430-07.

17. Kulasekara, B.R., Kamischke, C., Kulasekara, H.D., Christen, M., Wiggins, P.A., and Miller, S.I. (2013). c-di-GMP heterogeneity is generated by the chemotaxis machinery to regulate flagellar motility. eLife *2*, e01402. https://doi.org/10.7554/eLife.01402.
