# Supplementary figures and images for: Pseudomonads coordinate innate defense against viruses and bacteria with a single regulatory system
Source: bioRxiv. 2025 Feb 27:2025.02.26.640152. Preprint. [Version 1] doi: 10.1101/2025.02.26.640152 (PMC11888443; doi:10.1101/2025.02.26.640152)

Figure S1

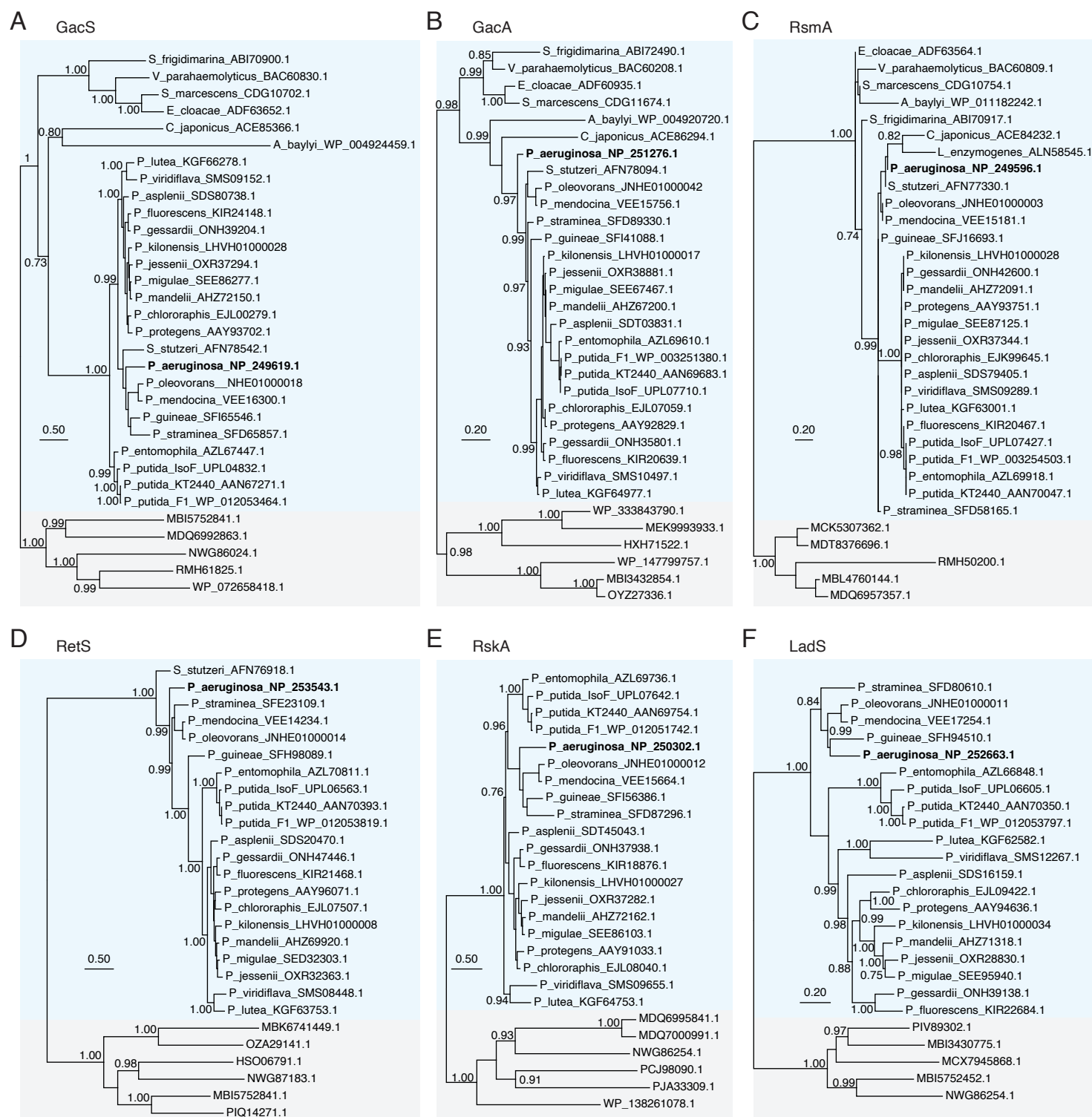

Figure S2

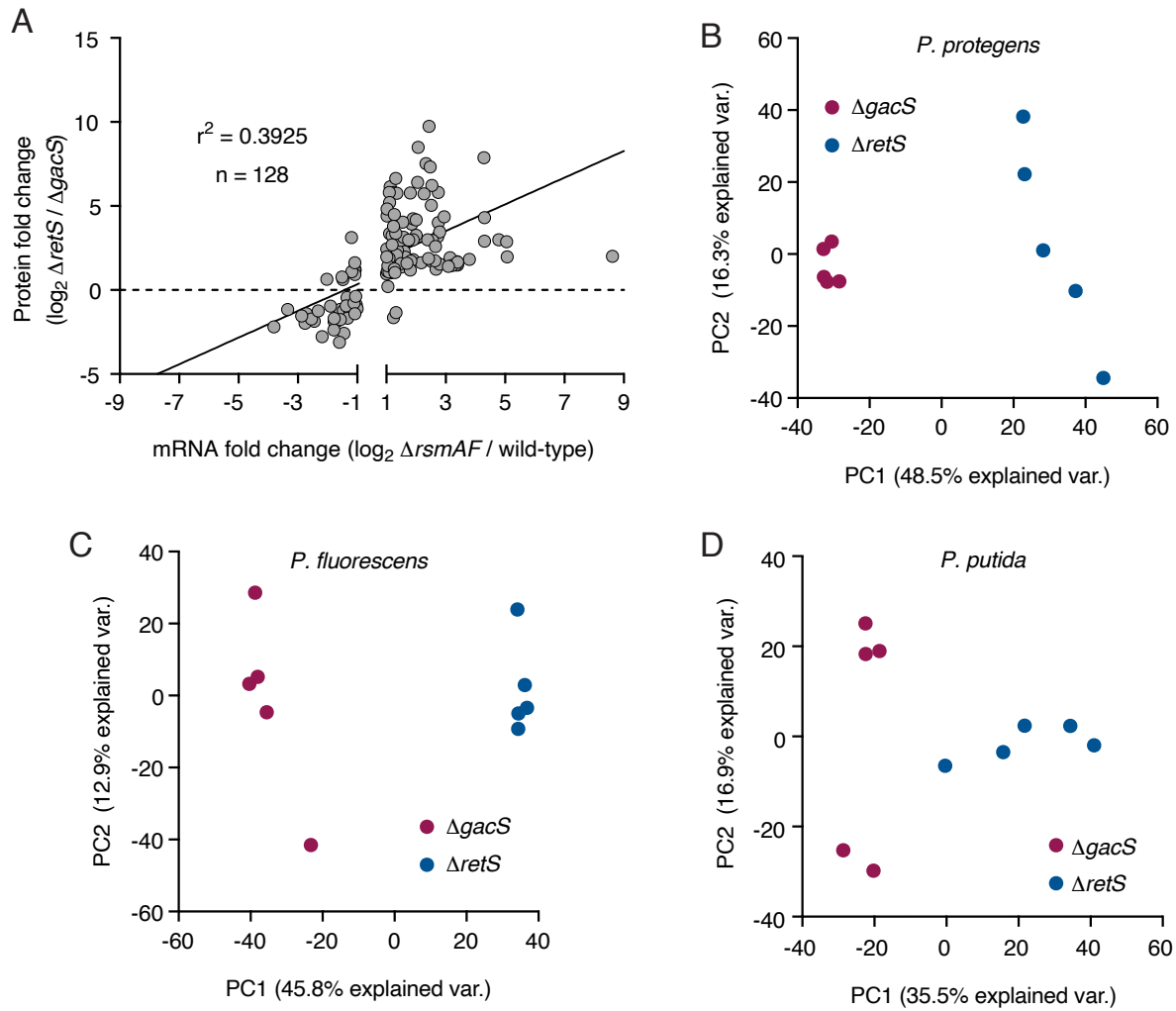

Figure S3

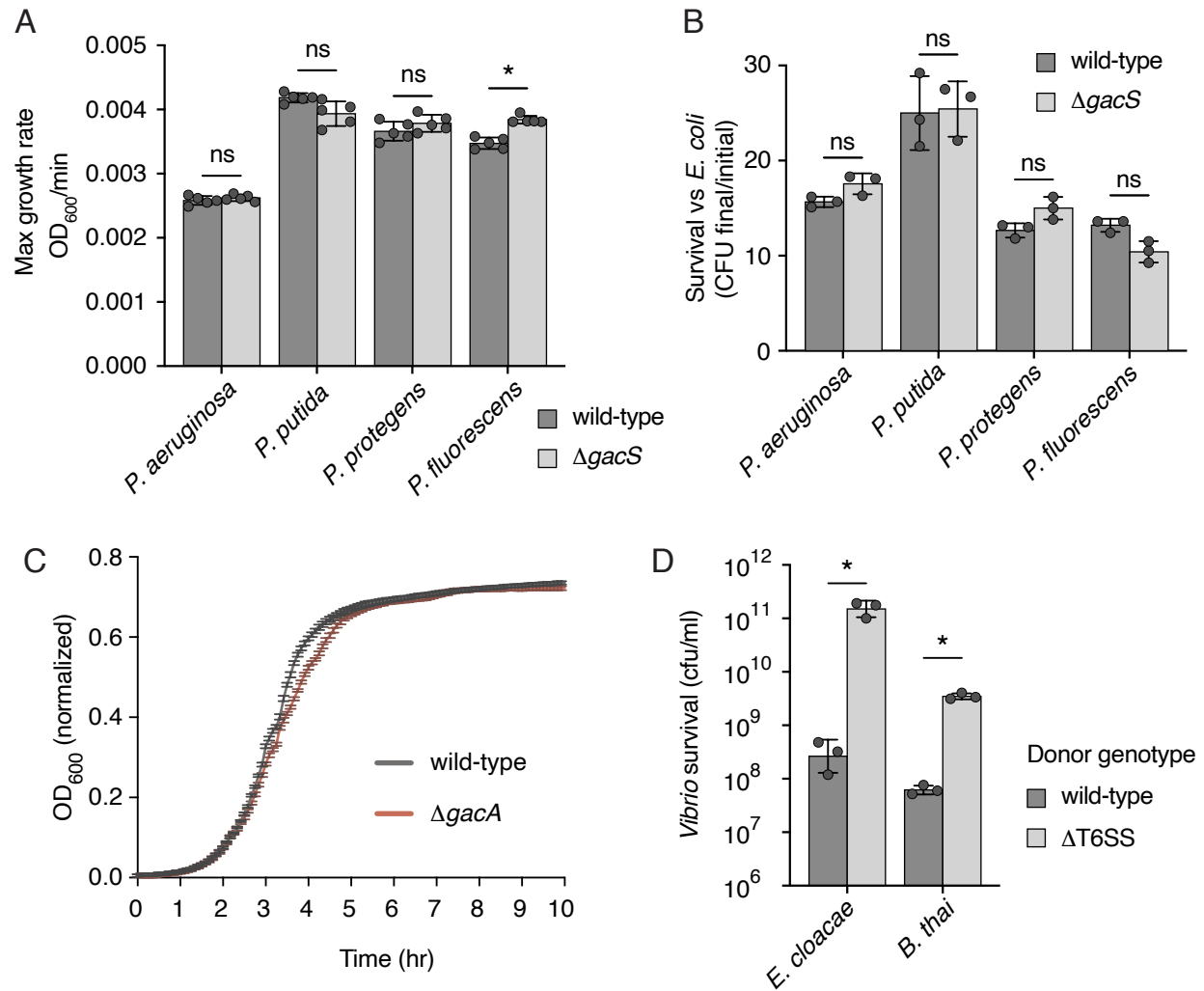

Figure S4

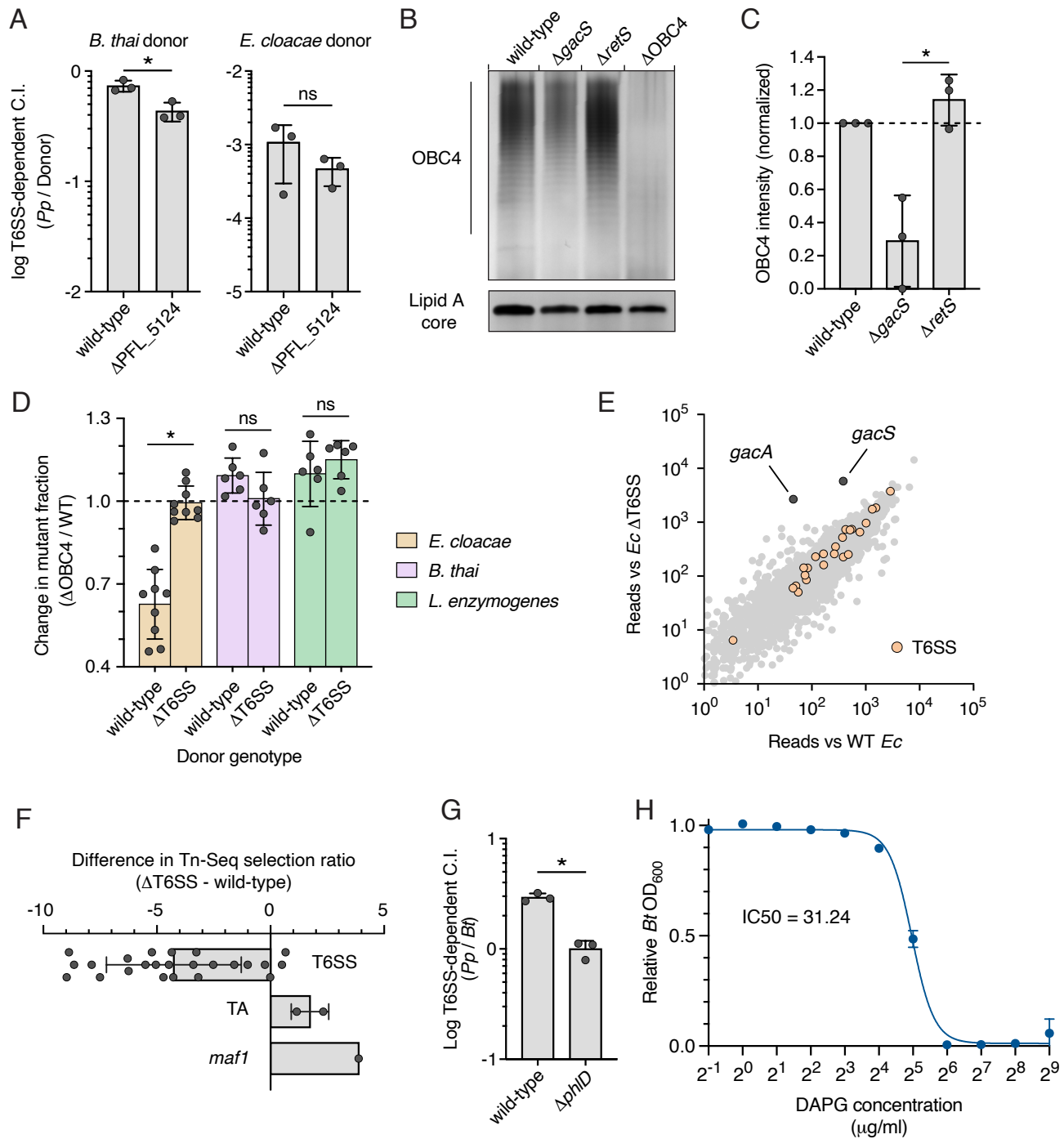

Figure S5

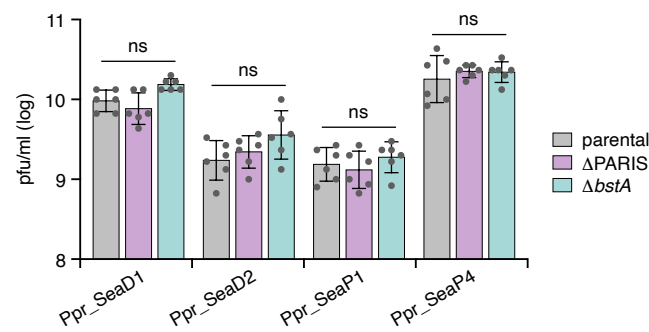

Supplement: 1 — Figure S1. Phylogenetic analysis enables confident identification of GRP signaling protein homologs. (A-F) Maximum-likelihood inferred phylogenies of homologs of the indicated GRP proteins from P. aeruginosa. Blue shading indicates orthologous proteins; more distant homologs used as outgroups indicated in grey. Figure S2. GRP activation yields a replicable shift in the proteomes of Pseudomonas species. (A) Comparison of the fold change of protein between P. aeruginosa ΔretS and ΔgacS (this study) with transcript fold change between P. aeruginosa ΔrsmAF and wild-type from a published RNA-seq dataset32. (B-D) Principal component analysis of MS-derived whole-cell proteomes (n=5) from the indicated strains of P. protegens (B), P. fluorescens (C), and P. putida (D). Figure S3. The GRP is dispensable in the absence of antagonism. (A) Comparison of maximum growth rate, defined as the largest change in OD600 in a 5-minute interval, between wild-type and gacS mutant strains of the indicated Pseudomonas species (n=5). (B) Survival of wild-type and gacS mutant strains of the indicated Pseudomonas species in competition with the non-antagonistic E. coli strain MG1655 (n=3). (C) Liquid growth curve measuring OD600 of wild-type V. parahaemolyticus and a gacA mutant strain over time (n=10). OD600 is normalized to a blank LB well, and error bars indicate SD. (D) Survival of V. parahaemolyticus in competition with the indicated strains of E. cloacae and B. thai, determined by recovered cfu concentrations (n=3). Statistical significance for all panels was determined using a Welch’s t-test with BH correction for multiple comparisons. Figure S4. GRP-regulated factors protect against antagonism and their effect can be masked by the T6SS. (A) Competitive fitness of P. protegens lacking periplasmic protease PFL_5124 in response to T6SS antagonism by B. thai or E. cloacae (n=3). (B) Representative LPS profiles of the indicated P. protegens strain, normalized by OD600, run on an 8–16% gradi [file NIHPP2025.02.26.640152V1-supplement-1.pdf]
